# Multidimensional pain assessment and opioid use after total knee arthroplasty: continuous vs single-injection regional vs systemic analgesia

**DOI:** 10.1097/PR9.0000000000001257

**Published:** 2025-03-18

**Authors:** Michael A. Harnik, Oskar Oswald, Markus Huber, Debora M. Hofer, Marcus Komann, Johannes Dreiling, Ulrike M. Stamer

**Affiliations:** a Department of Anaesthesiology and Pain Medicine, Inselspital, Bern University Hospital, University of Bern, Bern, Switzerland; b Department of Anaesthesiology, Intensive Care, Emergency and Pain Medicine, Centre for Interdisciplinary Pain Medicine, University Hospital Würzburg, Würzburg, Germany; c Department of Anesthesiology and Intensive Care Medicine, Jena University Hospital, Friedrich Schiller University Jena, Jena, Germany; d Department for BioMedical Research, University of Bern, Bern, Switzerland

**Keywords:** Total knee arthroplasty, Peripheral nerve block, Continuous peripheral nerve block, Postoperative pain management, Opioid use, Assessment of pain/pain assessment, Pain-related patient-reported outcomes, Multidimensional pain scores

## Abstract

**Introduction:**

Effective pain management after total knee arthroplasty (TKA) is essential for recovery. Continuous peripheral nerve blocks (PNBc) are often believed to provide superior pain relief compared with single-injection peripheral nerve blocks (PNBs). However, multidimensional pain-related patient-reported outcomes (PROs) have not been extensively studied.

**Objective:**

Based on registry data, this study compared pain intensities summarized as a pain composite score (PCS) and postoperative opioid use between PNBc and PNBs nerve blocks in patients undergoing TKA, and evaluated additional PROs.

**Methods:**

Data from 4,328 adults undergoing TKA enrolled in the PAIN OUT registry (ClinicalTrials.gov NCT02083835) were analyzed. Patients were categorized into general anesthesia (GA) or spinal anesthesia (SA), with subgroups general anesthesia only (GA-o) or spinal anesthesia only (SA-o), and combinations with single-injection PNB (GA&PNBs and SA&PNBs) or continuous PNB via catheter (GA&PNBc and SA&PNBc). The primary end point was PCS, summarizing pain intensities and time in severe pain during the first 24 hours. Secondary end points included opioid use and additional PROs.

**Results:**

The use of GA&PNBc was associated with a higher PCS (+0.5 [0.0-0.9], *P* = 0.035) compared with GA&PNBs, while PCS was similar between SA&PNBs and SA&PNBc. Opioid use was more frequent in GA&PNBc (+20.3%) and SA&PNBc (+50.8%) compared with the respective PNBs groups (*P* < 0.001). Patient-reported outcomes were higher in PNBc groups (median score 3.2 vs 2.7-2.9 in other groups; *P* < 0.001).

**Conclusion:**

Continuous PNBc showed no clear advantage over PNBs in pain relief, opioid use, or further PROs. Future research should incorporate comprehensive PROs to better evaluate analgesic techniques in TKA.

## Abstract 2

Supplemental Digital Content is Available in the Text.

Continuous nerve blocks for TKA offered no significant advantage over single-injection blocks in pain relief, opioid use, or patient-reported outcomes during the first postoperative day.

## 1. Introduction

Total knee arthroplasty (TKA) is a major orthopedic intervention frequently performed worldwide. Optimal postoperative pain control is essential to enable timely mobilization, rapid return to sufficient function, and prevention of pain chronification.^15,18^ Nevertheless, up to 20% of patients report chronic postsurgical pain after TKA, which may also result in prolonged opioid use, emphasizing the importance of effective perioperative analgesia.^58^

Previous publications and guidelines have advocated the combination of peripheral nerve blocks (PNBs) with general anesthesia (GA) or exclusive reliance on regional anesthesia for TKA.^17,20,35,36^ Notably, some studies have posited that continuous peripheral nerve blocks (PNBc) might offer superior outcomes compared with single-injection techniques (PNBs), resulting in more efficacious pain reduction and/or diminished postoperative opioid use.^3,7,26,29,43,45^ However, only unidimensional measures of pain intensity, such as “maximum” or “average” pain scores have often been used.^7,23,45^ Meanwhile, the multidimensional aspects of “pain severity” are underlined: pain should no longer be assessed using a unidimensional pain scale only, but using a more comprehensive approach, reflecting the subdomains *pain intensity*, *pain-related functional* and *emotional interferences*, and *pain-related adverse events*.^19,21,28,37,47,61^

The present registry analysis aimed to compare the influence of PNBc with PNBs and anesthetic techniques without PNB on (1) a pain composite score (PCS) of postoperative pain intensity and time in severe pain, (2) the proportion of patients requiring postoperative opioids, and (3) further pain-related patient-reported outcomes (PROs), reflecting *pain severity*. Our hypothesis was that GA&PNBc and spinal anesthesia SA&PNBc may result in reduced pain intensity in fewer patients receiving additional opioids during the first 24 hours postoperatively and in less pain severity compared with the other anesthesia groups.

## 2. Methods

### 2.1. Study design

The analysis included registry data retrieved from the international quality improvement repository PAIN OUT (ClinicalTrials.gov NCT02083835), originally funded by the European Union. Details have been published before^25^: PAIN OUT inclusion criteria were age of at least 18 years, patients' informed consent, and patients on their first postoperative day staying in the ward for at least 6 hours. Exclusion criteria were inability to communicate (such as being asleep or too tired to answer the questionnaire), cognitive impairment, or refusal to participate in the study.

On the first postoperative day, patients completed the validated PAIN OUT questionnaire on PROs.^25,41,42,61^ They rated their pain intensity, pain-related functional interference, adverse events, and perception of care for the first 24 hours after surgery using the numeric rating scale (NRS 0–10, 0 = no pain, no interference, and no symptoms; 10 = worst pain imaginable, pain completely interfered with function, and severe symptoms), yes/no answers, or the percentage scale (questionnaire available on the PAIN OUT homepage^25^). Patient characteristics (age, sex, weight, height, and countries grouped by income), pre-existing chronic pain, pre-existing opioid medication, and/or substance abuse (ie, abuse of drugs, legal and illegal) were documented according to patients' charts and/or self-reports. Furthermore, the details of anesthesia as well as analgesia in the postanesthesia care unit and ward were retrieved from patients' charts. Data were automatically coded and entered into a password-secured web-based repository. This study adhered to the RECORD guidelines.^4^

### 2.2. Study population

After acceptance of the study plan by the PAIN OUT publication board, ethics approval was obtained for the analysis of the registry data (Kantonale Ethikkommission Bern, BASEC 021-01878). The PAIN OUT board provided 4759 data sets of patients who underwent TKA (International Classification of Diseases, Ninth Revision code 81.54) between January 2010 and March 2020. Based on exclusion rates reported in previous studies, we expected an adequate sample size of at least 100 patients per group.^8,13,29,39^ Entries were checked for plausibility (age of at least 18 years, weight ≥34 kg, and the range of administered analgesic doses). Only the data sets with complete variables for the primary outcome were included.

### 2.3. Anesthesia groups

Patients were allocated to the GA or SA groups. Further subgroups were differentiated with or without PNBs (femoral, adductor canal, sciatic block, or a combination of both). This resulted in the following anesthesia subgroups:

(1) GA-o: general anesthesia only.
(2) GA&PNBs: general anesthesia and PNB single-injection.
(3) GA&PNBc: general anesthesia and continuous PNB via catheter technique used for postoperative analgesia in the ward.
(4) SA-o: spinal anesthesia only.
(5) SA&PNBs: spinal anesthesia and PNB single-injection.
(6) SA&PNBc: spinal anesthesia and continuous PNB via catheter technique used for postoperative analgesia in the ward.

### 2.4. Pain composite score

As a unidimensional assessment of pain is no longer recommended, composite scores have gained increasing recognition in pain research because they can capture multiple aspects of pain outcomes in one variable.^19,53^ The PAIN OUT registry comprises multidimensional reported outcomes reflecting the patients' perspective and adheres to current recommendations for assessing acute postoperative pain.^27^ *Pain intensity* was evaluated using a PCS of *maximum pain*, *minimum pain*, and % of *time in severe pain* during the first 24 hours after surgery. The PCS combines these measures, resulting in a NRS score of 0 to 10, as previously published (Calculation PCS: sum of NRS maximal pain × time in severe pain + NRS least pain × [1 − time in severe pain]) (see example in the Supplemental Content, Eq. (A.1), available at http://links.lww.com/PR9/A290).^22,48^ In particular, including the *time in severe pain* as a meaningful variable is important, as its relevance has been underlined in clinical research.^48,51^ The PCS was the primary end point, which was compared between the anesthesia groups and subgroups. Further comparisons were made between patients with and without preoperative opioid medication and/or substance use (subcohort O&S with substance abuse of legal and/or illegal drugs).

### 2.5. Secondary end points

Furthermore, the dichotomous variables of opioid use (yes/no) and administered opioid doses during the first 24 hours after surgery, calculated as morphine equivalents (MEs) in milligrams using standard conversion factors, were recorded.^38^ Secondary end points addressed the other subdomains of *pain severity* (*pain-related physical and emotional interference* and *adverse events related to pain management*) to capture patients' overall postoperative pain experience more comprehensively. Based on this, the corresponding composite scores (NRS 0-10) were calculated: pain-related physical interference total score (PITS), pain-related emotional interference composite score (EIS), and adverse events composite score (AES). A multidimensional PRO score (PRO-Score) summarizes all composite scores (PCS, PITS, EIS, and AES) as a global measure of *pain severity*.

To assess perception of care, we analyzed patients' responses considering their desire for more treatment (“Would you have liked MORE pain treatment than you received?”) and their satisfaction with pain treatment during the first 24 hours after surgery. Furthermore, the percentage of patients receiving nonopioid analgesics before the end of surgery or postoperatively was determined. In addition, the origin of data was determined and categorized into high vs middle-/low-income countries according to World Bank Classification. Three consecutive periods of enrolment were included as covariates to control for changes in practice over time: 2010 to 2012, 2013 to 2017, and 2017 to 2020.

### 2.6. Statistical analysis

Categorical variables are presented as n (%). Unadjusted group comparisons of categorical variables were performed using the Pearson χ^2^ test. For continuous variables and ordinal data (NRS scores), medians with interquartile ranges (first/third quartile [Q1; Q3]) were calculated. For exploratory purposes, unadjusted group comparisons of continuous variables were computed using an analysis of variance, the Mann–Whitney *U*, or the Kruskal–Wallis test.

The primary end point (PCS) was analyzed using a multivariable fixed-effects regression model with a beta-distributed outcome. We rescaled the observed PCS (NRS 0-10) by multiplying it by 0.1 to fit within the (0, 1) interval.^46^ The transformed PCS outcome served as the dependent variable in the model assessing the association between anesthesia techniques and patient characteristics, considering the beta distribution of the PCS values. Fixed effects included the anesthesia groups, sex, age, weight, year of survey (categorized), and preoperative or intraoperative and postoperative administration of nonopioid analgesics, opioids in the postanesthesia care unit and on the ward (see Supplemental Table T1, available at http://links.lww.com/PR9/A290). To account for similarities or possible differences in treatment strategies between high- and middle-/low-income countries, a random offset was included in the model. The adjusted estimated marginal means with 95% CI and pairwise contrasts were modeled for the GA and SA groups and all anesthesia subgroups and then further split into cases with and without pre-existing opioid medication or substance use (yes/no). Pairwise contrasts were adjusted using the Tukey range test. The number of data points included is presented in detail in each table. No imputation was performed, and each step in the analysis was based on a complete case analysis.

To assess the relationship between the percentage of patients receiving opioids and anesthesia group, an additional multivariable logistic regression model was created. GA-o was chosen as the reference category for anesthesia techniques. The same covariates were included as in the primary analysis (see Supplemental Table T2, available at http://links.lww.com/PR9/A290). Odds ratios and their respective 95% confidence intervals were reported. For further outcomes (composite scores of PROs), unadjusted group comparisons were applied to categorical variables, including the desire for more treatment, satisfaction, allowed to participate, pain relief, and treatment information received, using the Mann–Whitney *U* test for 2 groups or the Kruskal–Wallis test for multiple groups. Categorical variables were analyzed using the Pearson χ^2^ test. All analyses were computed using R version 4.0.2. Statistical significance was set at *P* < 0.05.

## 3. Results

### 3.1. Study population and patient characteristics

Of the 4759 patients who underwent TKA, those with a combination of SA and GA were interpreted as having failed SA and were excluded (see Supplemental Figure S1, available at http://links.lww.com/PR9/A290). Patients who received a combination of general and epidural anesthesia were also excluded because it was not possible to determine whether they had received sedation or full narcosis with a secured airway. The analyzed population with complete variables for the primary outcome consisted of 4328 patients. Of these, 44.8% received GA and 55.2% had SA. In the anesthesia subgroups, most patients received SA-o (24.3%), whereas both PNBc groups comprised 31% of all patients (18.2% with GA&PNBc and 12.8% with SA&PNBc). The use of continuous PNB ranged from 0% to 54.9% in the top 10 contributing hospitals. In those cases, in which the optional information of the type of PNB was documented, femoral nerve blocks were the most commonly performed blocks, particularly in the GA group (50.9%; see Supplemental Table T3, available at http://links.lww.com/PR9/A290). Pre-existing chronic pain before surgery was frequent (88.8%; Table 1). The O&S subcohort consisted of 412 patients (9.5%), 17 of whom had substance abuse. Approximately three-quarters of the patients were from high-income countries, where most PNBc were performed across both GA and SA. By contrast, PNBc was rarely performed in middle-/low-income countries. Most frequently used techniques in these hospitals were SA-o, SA&PNBs, and GA&PNBs. Detailed patient characteristics are summarized in Table 1.

**Table 1:** Patient characteristics.

	Groups			Subgroups							
	GA (N = 1941)	SA (N = 2387)	*P*	GA-o (N = 589)	GA&PNBc (N = 789)	GA&PNBs (N = 563)	SA-o (N = 1051)	SA&PNBc (N = 556)	SA&PNBs (N = 780)	*P*	N
Sex			0.459							0.880	4319
Female	1230 (63.5)	1541 (64.7)		375 (63.8)	492 (62.5)	363 (64.7)	685 (65.2)	361 (65.0)	495 (63.7)		
Male	706 (36.5)	842 (35.3)		213 (36.2)	295 (37.5)	198 (35.3)	366 (34.8)	194 (35.0)	282 (36.3)		
Age (y)	67 [60; 74]	67 [61; 74]	0.301	68 [60; 74]	68 [60; 75]	66 [59; 73]	68 [62; 75]	65 [59; 73]	67 [61; 74]	0.002	4328
Weight (kg)	80 [69; 93]	79 [68; 92]	0.039	80 [67; 92]	81 [70; 94]	80 [67; 92]	78 [68; 90]	82 [70; 100]	77 [65; 89]	<0.001	4328
Pre-existing pain	1644 (87.6)	2084 (89.8)	0.031	490 (87.5)	684 (88.0)	470 (87.0)	917 (89.6)	491 (91.3)	676 (88.9)	0.223	4199
Pre-existing pain intensity (NRS 0-10)	7 [5; 8]	7 [5; 9]	<0.001	7 [5; 8]	7 [5; 8]	7 [5; 8]	7 [6; 9]	7 [5; 8]	7 [6; 9]	<0.001	4328
Pre-existing pain location			0.152							0.503	3664
Site of surgery	1152 (71.2)	1395 (68.2)		350 (72.2)	464 (68.7)	338 (73.6)	623 (69.1)	322 (67.2)	450 (67.8)		
Elsewhere	38 (2.35)	56 (2.74)		11 (2.27)	18 (2.67)	9 (1.96)	28 (3.10)	11 (2.30)	17 (2.56)		
Both	429 (26.5)	594 (29.0)		124 (25.6)	193 (28.6)	112 (24.4)	251 (27.8)	146 (30.5)	197 (29.7)		
Opioids + substances	209 (10.8)	203 (8.50)	0.013	58 (9.85)	85 (10.8)	66 (11.7)	103 (9.80)	35 (6.29)	65 (8.33)	0.026	4328
ME before surgery (mg)	30 [20; 60]	30 [20; 40]	0.009	30 [15; 61]	31 [20; 60]	40 [16; 60]	30 [20; 40]	20 [10; 30]	30 [17; 45]	0.073	235
Duration surgery (min)	115 [90; 144]	97 [79; 120]	<0.001	117 [94; 144]	120 [95; 145]	107 [80; 137]	100 [82; 134]	98 [80; 121]	93 [73; 115]	<0.001	3947
High-income country	1505 (46.5)	1732 (53.5)	<0.001	415 (12.8)	750 (23.2)	340 (10.5)	705 (21.8)	492 (15.2)	535 (16.5)	<0.001	4328
Low-/middle-income country	436 (40.0)	655 (60.0)	<0.001	174 (15.9)	39 (3.6)	223 (20.4)	346 (31.7)	64 (5.9)	245 (22.5)	<0.001	

Data are presented as median [Q1; Q3] or n (%). Statistical analyses: Mann–Whitney *U* test, Kruskal–Wallis test, and Pearson χ^2^ test.

GA&PNBc, general anesthesia and PNB catheter; GA&PNBs, general anesthesia and PNB single-injection; GA-o, general anesthesia only, no additional nerve block; ME, morphine equivalents (only for patients taking opioids); NRS, numeric rating scale; SA&PNBc, spinal anesthesia and PNB catheter; SA&PNBs, spinal anesthesia and PNB single-injection; SA-o, spinal anesthesia only.

### 3.2. Pain composite score

**Figure 1.**
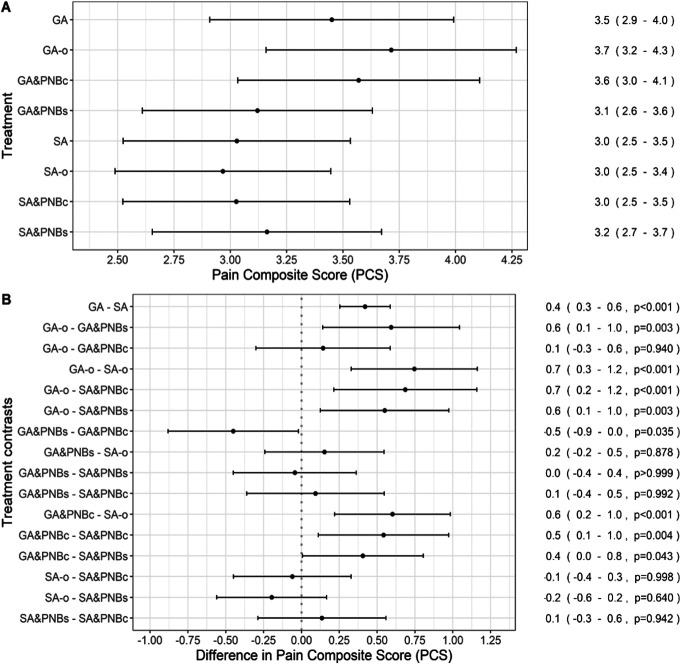
Adjusted estimated marginal means and adjusted pairwise contrasts for the PCS for groups and subgroups. (A) Adjusted estimated marginal means of the PCS for the anesthesia groups and the subgroups represented on the *y*-axis. The *x*-axis represents the NRS (0-10). Specific means and confidence intervals are provided on the right side. (B) Adjusted pairwise contrasts of PCS for all subgroups. Specific percentage differences, confidence intervals, and *P* values for each comparison are shown on the right. Statistical analyses were performed using a multivariable fixed-effects regression model with a beta-distributed outcome. A *P* value <0.05 was considered as statistically significant. GA, general anesthesia (all techniques); GA&PNBc, general anesthesia and PNB catheter used for postoperative analgesia; GA&PNBs, general anesthesia and PNB single-injection, with patients receiving a peripheral nerve block intraoperatively; GA-o, general anesthesia without an additional nerve block; NRS, numeric rating scale; PCS, pain composite score; SA&PNBc, spinal anesthesia and PNB catheter; SA&PNBs, spinal anesthesia and PNB single-injection; SA, spinal anesthesia (all techniques); SA-o, spinal anesthesia only.

The unadjusted median pain scores and composite scores are summarized in Table 2. For the adjusted estimated marginal means, all patients with SA techniques reported a PCS between NRS 3.0 and 3.2. The only GA technique with a comparably low PCS was GA&PNBs with 3.1 (2.6-3.6). The PCS of the other 2 GA groups ranged from 3.6 to 3.7 (Fig. 1A). No significant differences were observed in the pairwise comparisons among the SA groups and the GA&PNBs group (eg, the PCS for GA&PNBs compared with SA&PNBs was 0.0 [−0.4 to 0.4, *P* > 0.999]). Considering GA&PNBc, this group had a higher PCS than the GA&PNBs group (+0.5 [0.0-0.9, *P* = 0.035]; Fig. 1B).

**Table 2:** Patient-reported outcomes and analgesic use in the postanesthesia care unit and on the ward during the first postoperative day (n (%)) with doses in milligrams morphine equivalent for those who received opioids.

	Groups			Subgroups						
	GA (N = 1941)	SA (N = 2387)	*P*	GA-o (N = 589)	GA&PNBc (N = 789)	GA&PNBs (N = 563)	SA-o (N = 1051)	SA&PNBc (N = 556)	SA&PNBs (N = 780)	*P*
PCS	3.2 [1.3; 5.4]	2.8 [1.0; 5.0]	<0.001	3.2 [1.3; 5.4]	3.5 [1.6; 5.5]	2.8 [0.9; 5.0]	2.9 [1.0; 5.2]	2.8 [1.0; 5.0]	2.6 [1.0; 4.8]	<0.001
Worst pain (NRS)	6 [4; 8]	6 [4; 8]	0.402	7 [4; 8]	6 [4; 8]	6 [4; 8]	6 [4; 8]	6 [4; 8]	6 [4; 8]	0.152
Least pain (NRS)	2 [0; 3]	2 [0; 3]	0.064	2 [1; 4]	2 [1; 3]	2 [0; 3]	2 [0; 3]	2 [0; 3]	2 [0; 3]	0.277
% Time in severe pain	30 [10; 50]	20 [10; 50]	<0.001	20 [10; 50]	30 [10; 50]	20 [10; 50]	20 [10; 50]	20 [10; 40]	20 [0; 50]	<0.001
PITS	2.3 [1.0; 4.0]	2.3 [1.0; 4.0]	0.008	2.3 [1.0; 3.6]	2.7 [1.3; 4.3]	2.3 [1.0; 4.0]	2.3 [0.7; 4.0]	2.3 [1.0; 3.7]	2.3 [1.0; 4.0]	0.002
EIS	1.0 [0.0; 4.0]	1.5 [0.0; 4.0]	0.083	1.0 [0.0; 4.0]	1.5 [0.0; 4.5]	0.5 [0.0; 3.5]	1.0 [0.0; 4.0]	2.0 [0.0; 4.0]	1.5 [0.0; 4.0]	<0.001
Helplessness (any)	899 (46.3)	1161 (48.6)	0.134	250 (42.4)	406 (51.5)	243 (43.2)	248 (43.6)	318 (57.2)	385 (49.4)	<0.001
Anxiety (any)	1000 (51.5)	1321 (55.3)	0.013	293 (49.7)	439 (55.6)	268 (47.6)	563 (53.6)	341 (61.3)	417 (53.5)	<0.001
AES	1.0 [0.3; 2.5]	1.3 [0.0; 2.8]	0.334	1.0 [0.0; 2.5]	1.0 [0.3; 2.5]	1.0 [0.3; 2.5]	1.3 [0.0; 2.8]	1.3 [0.3; 2.8]	1.1 [0.0; 2.5]	0.374
PRO-Score	2.9 [1.5; 5.0]	2.9 [1.4; 4.9]	0.042	2.8 [1.5; 4.9]	3.2 [1.8; 5.2]	2.7 [1.3; 5.0]	2.9 [1.3; 4.9]	3.2 [1.6; 4.9]	2.8 [1.3; 4.8]	<0.001
Preoperative/intraoperative nonopioids (yes)	1249 (64.3)	969 (40.6)	<0.001	368 (62.5)	456 (57.8)	425 (75.5)	305 (29.0)	150 (27.0)	514 (65.9)	<0.001
Postoperative nonopioids (yes)	1829 (94.2)	2220 (93.0)	0.106	552 (93.7)	758 (96.1)	519 (92.2)	958 (91.2)	523 (94.1)	739 (94.7)	<0.001
Postoperative opioids (yes)	1831 (94.3)	2083 (87.3)	<0.001	546 (92.7)	783 (99.2)	502 (89.2)	903 (85.9)	553 (99.5)	627 (80.4)	<0.001
Postoperative ME (mg)	18 [10; 32]	14 [7; 28]	<0.001	19 [10; 33]	18 [10; 30]	17 [9; 33]	18 [7; 30]	14 [7; 25]	10 [5; 22]	<0.001
Patients without opioids and substances (n = 3066) (mg)	18 [9; 30]	13 [6; 27]	<0.001	19 [10; 32]	17 [9; 29]	17 [8; 32]	17 [6; 30]	14 [7; 25]	10 [5; 20]	<0.001
Patients with opioids and substances (n = 344) (mg)	23 [13; 44]	23 [11; 33]	0.159	22 [11; 44]	20 [11; 39]	26 [13; 50]	23 [13; 32]	15 [8; 33]	20 [13; 33]	0.332

Continuous and ordinal data are shown as median [Q1; Q3] and frequencies as n (%). Statistical analyses: Mann–Whitney *U* test, Kruskal–Wallis test, and Pearson χ^2^ test.

AES, adverse events score; EIS, emotional interference score; GA&PNBc, general anesthesia and PNB catheter; GA&PNBs, general anesthesia and PNB single-injection; GA-o, general anesthesia only, no additional nerve block; ME, morphine equivalents (only patients with postoperative opioids); PCS, pain composite score; PITS, pain-related interference total score; PRO-Score, multidimensional composite score summarizing PCS, PITS, EIS, AES; SA&PNBc, spinal anesthesia and PNB catheter; SA&PNBs, spinal anesthesia and PNB single-injection; SA-o, spinal anesthesia only.

**Figure 2.**
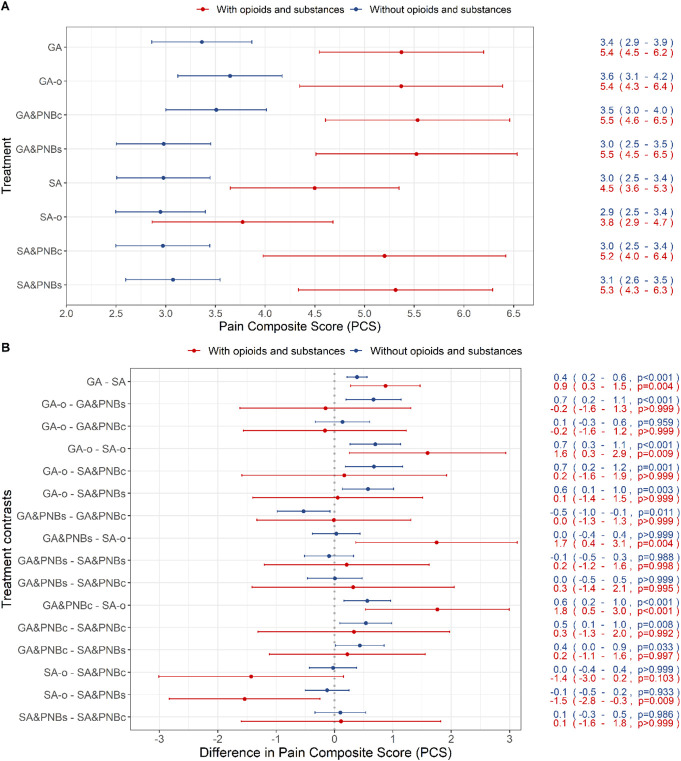
Adjusted estimated marginal means and adjusted pairwise contrasts for the PCS for patients without and with opioid or substance use before surgery. (A) Adjusted estimated marginal means of the PCS for the anesthesia groups and subgroups, represented on the *y*-axis and differentiated for patients with opioids and/or substances (red lines) and those without (blue lines). The *x*-axis represents the NRS (0-10). Specific means and confidence intervals are provided on the right. (B) Adjusted pairwise contrasts of the PCS with comparisons of all subgroups differentiated for patients with O&S (N = 412) and those without (N = 3916). Specific percentage differences, confidence intervals, and *P* values for each comparison are provided on the right. Statistical analyses were performed using a multivariable fixed-effect regression model with a beta-distributed outcome. A *P* value <0.05 was considered as statistically significant. GA, general anesthesia (all techniques); GA&PNBc, general anesthesia and PNB catheter; GA&PNBs, general anesthesia and PNB single-injection; GA-o, general anesthesia only, no additional nerve block; NRS, numeric rating scale; PCS, pain composite score; SA&PNBc, spinal anesthesia and PNB catheter; SA&PNBs, spinal anesthesia and PNB single-injection; SA, spinal anesthesia (all techniques); SA-o, spinal anesthesia only.

Patients with preoperative opioid medication or substance abuse consistently rated their postoperative pain as more severe than opioid-naïve patients (Fig. 2A). The lowest PCS in these patients was reported in the SA-o group (NRS: 3.8 [2.9-4.7]). In the pairwise contrasts, the SA-o group of the subcohort O&S had lower PCS of −1.5 to −1.8 compared with the groups GA-o, GA&PNBs, GA&PNBc, and SA&PNBs (all *P* < 0.01; Fig. 2B).

### 3.3. Postoperative opioid use

**Figure 3.**
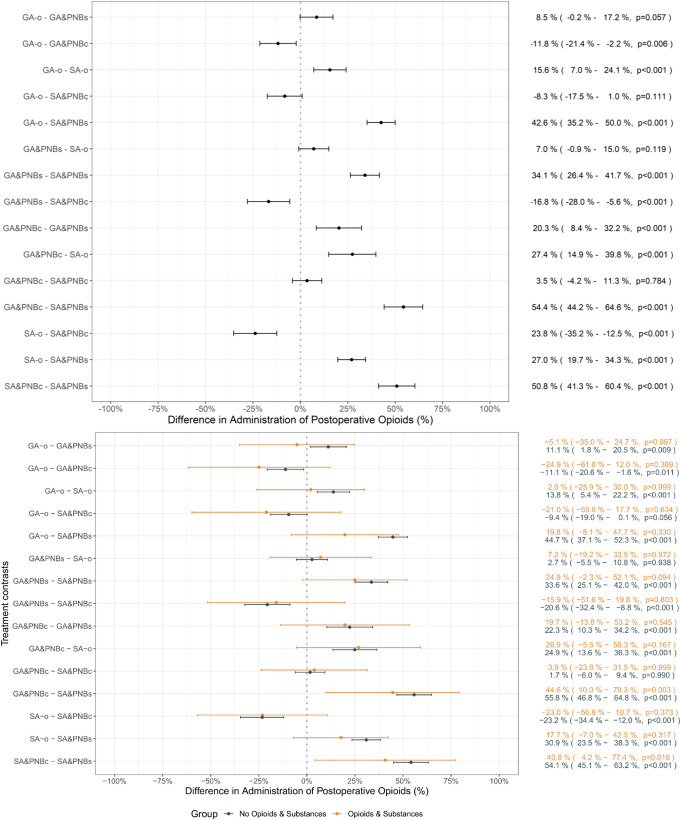
Treatment contrasts for % of patients receiving postoperative opioids across anesthesia techniques for the entire population and for patients without and with opioid or substance use before surgery. (A) Treatment contrasts of anesthesia groups of the entire population. Each line represents a treatment contrast with the mean percentage difference and the associated 95% confidence intervals. The *x*-axis displays the difference in the percentage of patients with opioid use, with 0% indicating no difference among the groups. Specific percentage differences, confidence intervals, and *P* values for each comparison are provided on the right. (B) Treatment contrasts of anesthesia groups of the 2 subcohorts with (N = 412) or without (N = 3916) preoperative O&S. Statistical analyses were performed by using a multivariable logistic regression model. GA&PNBc, general anesthesia and PNB catheter; GA&PNBs, general anesthesia and PNB single-injection; GA-o, general anesthesia only, no additional nerve block; SA&PNBc, spinal anesthesia and PNB catheter; SA&PNBs, spinal anesthesia and PNB single-injection; SA-o, spinal anesthesia only.

More than 80% of the entire study population received opioids postoperatively, with almost all patients in the GA&PNBc and SA&PNBc groups (>99%, Table 2). Consequently, in the GA&PNBc group, the pairwise treatment contrasts revealed that +11.8% of patients were treated with opioids compared with GA-o (*P* = 0.006) and +20.3% compared with GA&PNBs (*P* < 0.001; Fig. 3A). Similarly, in group SA&PNBc, +50.8% more patients received opioids postoperatively compared with SA&PNBs and +23.8% compared with SA-o (both *P* < 0.001). In subcohort O&S, patients received opioids more frequently in both PNBc groups (GA&PNBc: +44.6%, SA&PNBc: +40.8%) compared with SA&PNBs (Fig. 3B). Moreover, patients with O&S exhibited higher ME than those without preoperative opioid/substance intake (23 [13; 37] vs 15 [8; 29] mg, *P* < 0.001). No differences in postsurgical opioid doses were observed between the anesthesia groups in the O&S subcohort (Table 2). Conversely, patients without preoperative opioid or substance use displayed lower ME in the SA vs GA groups, with the lowest opioid doses in the SA&PNBs group (Table 2).

### 3.4. Further patient-reported outcomes

The multidimensional PRO-Score, summarizing subdomains of pain intensity, pain-related physical and emotional interference, and adverse events into a composite score for pain severity, ranged around a median NRS of 2.7 for GA&PNBs, 2.8 for SA& PNBs, and 3.2 for GA&PNBc and SA&PNBc, respectively (Table 2). The higher measures for the 2 PNBc groups resulted from differences in emotional interference; patients with continuous nerve blocks reported more emotional interference with an EIS of NRS 1.5 to 2.0. More emotional interference in the catheter groups arose from greater helplessness and anxiety in these patients (Table 3). Regarding pain-related physical interference, most patients reported a comparable PITS (NRS 2.3). The only exception were patients in group GA&PNBc, who indicated an increased PITS of 2.7 ([1.3; 4.3]; *P* = 0.002).

**Table 3:** Patients' perception of care.

	Groups			Subgroups							
	GA (N = 1858)	SA (N = 2290)	*P*	GA-o (N = 564)	GA&PNBc (N = 768)	GA&PNBs (N = 529)	SA-o (N = 1017)	SA&PNBc (N = 533)	SA&PNBs (N = 757)	N	*P*
Desire for more treatment (yes)	355 (19.1)	550 (23.9)	0.001	111 (19.9)	138 (18.0)	106 (20.0)	259 (25.4)	99 (18.8)	192 (25.5)	4154	<0.001
Satisfaction (NRS)	9 [7; 1]	9 [7; 10]	0.028	9 [7; 10]	9 [7; 10]	9 [7; 10]	9 [7; 10]	9 [7; 10]	9 [7; 10]	4097	0.255
Allowed to participate (NRS)	8 [4; 10]	8 [2; 10]	0.004	8 [2; 10]	8 [5; 10]	8 [2; 10]	7 [1; 10]	8 [5; 10]	7 [1; 10]	3990	<0.001
Pain relief (%)	70 [50; 90]	70 [50; 90]	0.009	70 [50; 90]	70 [50; 80]	70 [50; 90]	70 [50; 90]	80 [50; 90]	70 [50; 90]	3968	0.017
Treatment info received (yes)	1361 (73.3)	1616 (70.6)	0.061	357 (63.3)	629 (81.9)	375 (71.3)	674 (66.3)	435 (82.2)	507 (68.1)	4148	<0.001

Data are shown as n (%) or median [Q1; Q3]. Mann–Whitney *U* test, Kruskal–Wallis test, and Pearson χ^2^ test.

GA&PNBc, general anesthesia and PNB catheter; GA&PNBs, general anesthesia and PNB single-injection; GA-o, general anesthesia only, no additional nerve block; NRS, numeric rating scale; SA&PNBc, spinal anesthesia and PNB catheter; SA&PNBs, spinal anesthesia and PNB single-injection; SA-o, spinal anesthesia only.

Patients in the SA-o and SA&PNBs groups expressed a desire for additional pain treatment more often than those in the other subgroups (Table 3). By contrast, subgroups with higher rates of additional opioid administration, notably both PNBc groups, showed less desire for more treatment. No differences were observed in patient satisfaction.

## 4. Discussion

In the present analysis of registry data, PNBc combined with SA or GA did not result in lower PCS, reduced opioid use, or improvement in most other PROs compared with PNBs during the first 24 hours after surgery. Patients with O&S consistently rated their postoperative pain higher than those without.

### 4.1. Pain composite score

The primary outcome of this study was a composite measure of *pain intensity*, incorporating several variables, notably *time in severe pain*, which has been previously associated with chronic postsurgical pain.^22,48^ In stakeholder interviews, patients ranked *time in severe pain* and *worst pain* as the 2 most important pain indices.^51^ The PCS for the GA&PNBs group was comparable with that of the SA groups. By contrast, the use of PNBc did not result in a lower PCS, either in the entire study population or in the O&S subcohort. These findings suggest that anesthesia techniques may be interchangeable to some extent with respect to their impact on the composite score for *pain intensity*, with no significant advantage observed for PNBc within the first 24 hours postoperatively.

### 4.2. Continuous peripheral nerve blocks

For TKA, the effectiveness of PNBc remains debated, with some studies reporting superior pain relief, while others did not.^13,26,27,52,56,59^ In part, the less favorable results for PNBc in our investigation could be explained by different study settings: randomized controlled trials (RCTs) typically use stringent patient selection, exclude subjects with major comorbidities, ensure catheter placement by seasoned professionals, and provide rigorous postoperative care. The present analysis, derived from real-world data, hints at possible obstacles to consistently transferring the favorable results of some RCTs to clinical reality.

The limited use of PNBc in low- and middle-income countries compared with high-income countries is noteworthy. While the specific reasons remain unclear, restricted access to resources, such as catheter kits, may contribute to these differences.

### 4.3. Postoperative opioid use

Although opioids are frequently used in acute postoperative pain management, their side effects can lead to higher hospitalization costs, longer lengths of stay, respiratory depression, and higher death rates. Several investigations have demonstrated reduced opioid use in different orthopedic procedures when regional anesthesia was used.^2,9–11,40^ By contrast, when comparing PNBs and PNBc for adductor canal blocks, opioid doses used up to the second postoperative day did not differ in most RCTs.^16,33,34,54^ In the present investigation, the postoperative opioid doses differed among the anesthesia groups, but not in the O&S subcohort. Almost all patients with PNBc received opioids, challenging the assumption that PNBs are automatically opioid-sparing.^6,45^

The catheter groups did not have a higher proportion of patients with preoperative opioid/substance use. This speaks against the targeted use of PNBc in this well-described risk group. One potential explanation for patients with PNBc receiving opioids more frequently could be “rebound pain.”^12,49,50^ Moreover, proper patient education can aid the early recognition of increased pain after nerve block resolution or catheter failure.

### 4.4. Further patient-reported outcomes

Unlike previous studies, which often focused on single pain scores, the present investigation suggests how composite scores can be used in a multidimensional assessment of acute postoperative pain. The overall PRO-Scores reflects a measure of *pain severity* and provides a more comprehensive assessment based on previous suggestions on composite scores by Initiative on Methods, Measurement, and Pain Assessment in Clinical Trials.^5,24^ The decision to use composite scores acknowledges the need to capture the multifaceted nature of pain in one variable.^19^ However, further discussion in the pain community is necessary to obtain a consensus on accepted measures of multidimensional PROs.

Notably, patients who received PNBc had the highest PRO-Score and elevated levels of anxiety and helplessness, despite receiving the most extensive treatment information. This emotional subdomain could have been influenced by other factors such as constraints on movement imposed by an indwelling catheter. Regardless, the routine use of PNBc for TKA is no longer recommended because of reduced mobility due to muscle weakness, coupled with an increased risk of falls and overall inconsistent benefits.^32,57^

Conversely, patients with SA-o and SA&PNBs more frequently stated that they would have liked more pain treatment. This may be partially explained by fewer patients in these groups receiving additional opioids and less treatment information. Previous studies have demonstrated that the desire for more treatment is not solely influenced by pain intensity alone but also shaped by other factors, such as good communication between medical staff and patients, the amount of treatment information a patient receives, and being allowed to participate in decisions.^30^ Our results support this, as patients in these subgroups reported lower scores for participation in treatment decisions and received less information overall. In this regard, lower participation and less information could have led to increased desire for more treatment, and could reflect a lack of patient-centered care and deficient communication. All these components are considered essential for a personalized treatment approach and are associated with increased patient satisfaction.^44,60^ The potential association among the desire for more treatment, treatment information, and opioid administration was not anticipated in the a priori hypothesis. This aspect should be explored in future research.

### 4.5. Limitations and strengths of the study

Registry data share some common limitations. Although they provide important observations, generate meaningful associations, and serve as the basis for hypothesis generating, they cannot establish causal relationships. Selection bias cannot be ruled out, as clinicians may decide to use certain analgesic techniques according to patients' individual comorbidities and risk profiles (eg, the combination of SA and PNBc). The lower or nonexistent use of regional catheter techniques in low- and middle-income countries may be attributed to other reasons such as the lack of catheter sets for regional anesthesia. Not all confounders known to influence PROs were captured by this registry, such as psychosocial and socioeconomic aspects. Longer trajectories of outcomes may have revealed more pronounced differences between analgesia groups. Particularly for the comparison PNBs and PNBc, longer follow-up periods are needed to better assess multidimensional PROs over time. However, postoperative pain on the first day is associated with poorer long-term PROs and with chronic pain after surgery.^1,14,21,22,31,55^ Considering the substantial sample size of 4,328 patients, our investigation benefits from prospective, standardized data collection, providing valuable insights into real-world clinical practice. Finally, with the introduction of a composite score, we were able to assess the multidimensional aspects of postoperative pain after different anesthesia techniques. Although the PCS has been used previously, it has not been validated yet. Studies are required to validate the composite scores and assess the impact of the different pain subdomains.

### 4.6. Conclusions

Our results provide nuanced insights into patients' pain experiences by contrasting multidimensional PROs assessed during routine clinical practice for different anesthetic techniques. In the first 24 hours after surgery, the use of PNBc did not result in a more favorable PCS, a lower % of postoperative opioid application, or improvements in further PROs compared with PNBs. A broader perspective encompassing global PROs is warranted to evaluate the analgesic techniques for TKA.

## Disclosures

U. M. Stamer serves as the Subforum Lead for Acute and Chronic Pain and Palliative Medicine (2020-2023) within the European Society of Anaesthesiology and Intensive Care (ESAIC) and as the Lead of the Acute Pain Working Group for the German Pain Society. The other authors have no conflict of interest to declare.

## Appendix A. Supplemental digital content

Supplemental digital content associated with this article can be found online at http://links.lww.com/PR9/A290.
