# Supplementary material for: Multidimensional pain assessment and opioid use after total knee arthroplasty: continuous vs single-injection regional vs systemic analgesia
Source: Pain Rep. 2025 Mar 18;10(2):e1257. doi: 10.1097/PR9.0000000000001257 (PMC11922405; doi:10.1097/PR9.0000000000001257)
Supplement: Supplementary file 1 [file painreports-10-e1257-s001.pdf]

Supplementary materials

Eq. (A.1) Calculation of Composite Scores
PCS (pain composite score; NRS 0-10)): Sum of NRS maximal pain × time in severe pain + NRS least pain × (1 – time in severe pain) [23,49].
Example: NRS max =7, NRS min = 2, time in severe pain = 30% → 7 × 0.3 + 2 × (1-0.3) = 2.1 + 1.4 = 3.6

PITS (pain-related interference composite score): mean NRS 0-10) of pain-related interference in bed, interference with sleep, and breathing or coughing
EIS (emotional interference composite score): mean NRS (0-10) of pain-related anxiety and helplessness.
AES (adverse events composite score): mean NRS (0-10) of drowsiness, nausea, dizziness, and itching.
PRO-Score (Patient-Reported Outcomes Composite score): mean of PCS, PITS, EIS and AES.

Supplemental Figure S1: Flow chart according to the REporting of studies Conducted using Observational Routinely-collected Data (RECORD) statement

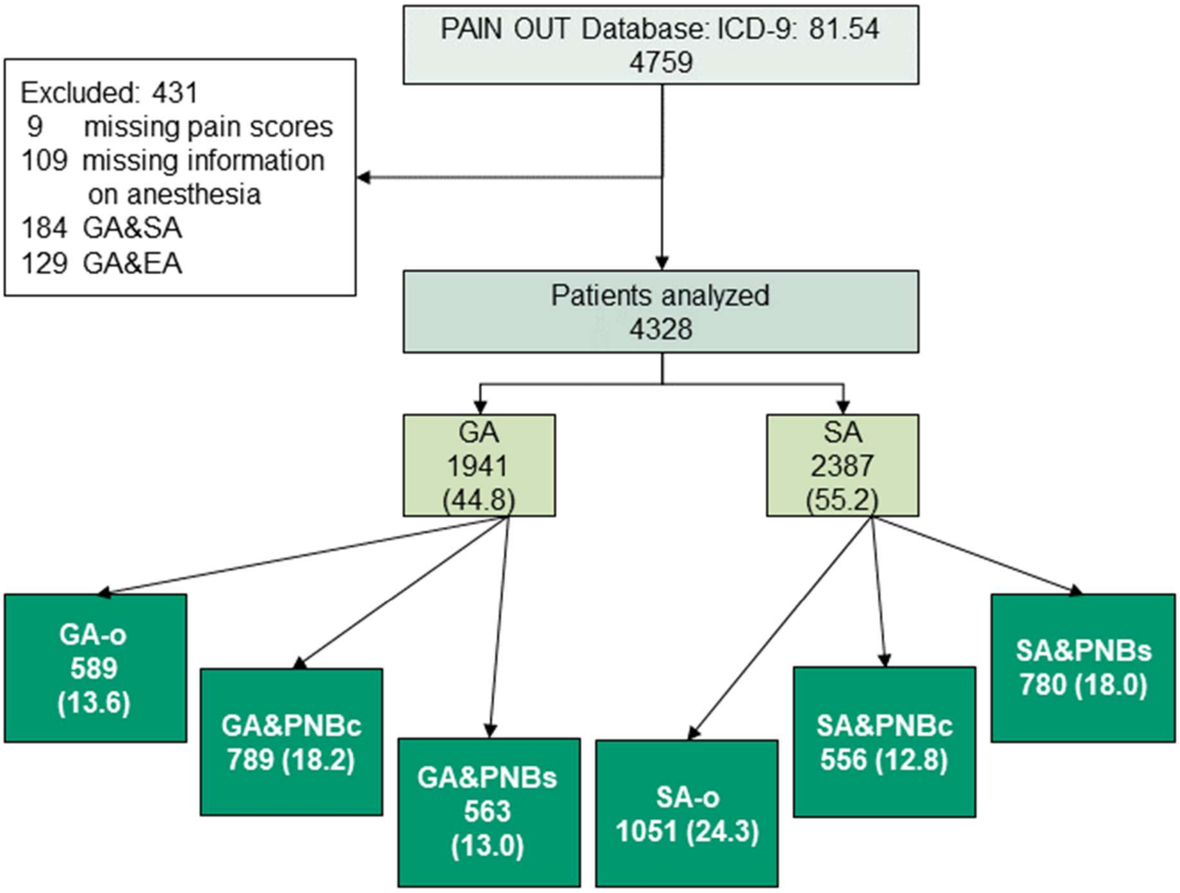

Number (%) of patients per anesthesia group who were enrolled and analyzed. GA-o, general anesthesia only; GA&PNBs, general anesthesia and PNB single-injection; GA&PNBc, general anesthesia and PNB catheter; SA-o, spinal anesthesia only; SA&PNBs, spinal anesthesia and PNB single-injection; SA&PNBc, spinal anesthesia and PNB catheter.

Supplemental Table T1: Regression coefficients for two multivariable regression models for the dependent variable PCS

	Groups		Subgroups	
**Characteristic**	**Beta (95% CI)**	***p***	**Beta (95% CI)**	***p***
**Anesthesia Groups**				
SA vs. GA	-0.19 (-0.27;-0.12)	<0.001		
GA&PNBs vs. GA-o			-0.27 (-0.40;-0.13)	<0.001
GA&PNBc vs. GA-o			-0.06 (-0.19;0.07)	0.354
SA-o vs. GA-o			-0.34 (-0.46;-0.21)	<0.001
SA&PNBs vs. GA-o			-0.25 (-0.37,-0.12)	<0.001
SA&PNBc vs. GA-o			-0.31 (-0.46,-0.16)	<0.001
**Age** (years)	-0.003 (-0.006;0.001)	0.14	-0.003 (-0.006;0.001)	0.125
**Sex:** males vs females	-0.15 (-0.23;-0.08)	<0.001	-0.16 (-0.23;-0.08)	<0.001
**Weight** (kg)	0.002 (0.000;0.004)	0.016	0.002 (0.001;0.004)	0.009
**Period of enrollment**				
2013-2017 vs. 2010-2012	-0.02 (-0.13;0.09)	0.7	-0.04 (-0.15;0.08)	0.530
2017-2020 vs. 2010-2012	-0.02 (-0.10;0.07)	0.7	-0.02 (-0.12;0.07)	0.618
**Preventive nonopioid analgesics** Yes vs. No	-0.10 (-0.18;-0.02)	0.013	-0.11 (-0.19;-0.02)	0.010
**Postoperative nonopioid analgesics** Yes vs. No	-0.21 (-0.36;-0.06)	0.006	-0.22 (-0.37;-0.07)	0.004
**Postoperative opioids** mg	0.004 (0.002;0.005)	<0.001	0.004 (0.002;0.005)	<0.001

One model features the GA and SA groups, whereas the other model explicitly resolves individual anesthesia subgroups. CI = Confidence Interval.

Supplemental Table T2: Logistic Regression Analysis: Comparison between anesthesia groups and other covariates regarding postoperative opioid use using odds ratios and 95% confidence intervals

Characteristic	OR	95% CI	*p*
**Anesthesia Group:**			
GA-o	—	—	
GA&PNBc	2.12	1.53, 2.92	<0.001
GA&PNBs	0.66	0.50, 0.87	0.003
SA-o	0.49	0.38, 0.62	<0.001
SA&PNBc	1.63	1.15, 2.30	0.006
SA&PNBs	0.15	0.12, 0.20	<0.001
**Age** (years)	0.99	0.99, 1.00	0.074
**Sex** (male)	0.78	0.67, 0.91	0.002
**Weight** (kg)	1.01	1.00, 1.01	<0.001
**Period of enrollment:**			
2010-2012	—	—	
2013-2017	1.48	1.15, 1.90	0.002
2017-2020	1.09	0.91, 1.31	0.362
**Preventive nonopioid analgesics** (yes)	0.67	0.57, 0.79	<0.001
**Postoperative nonopioid analgesics** (yes)	1.52	1.16, 2.01	0.003

OR = Odds Ratio, CI = Confidence Interval.

Supplemental Table T3: Type of peripheral nerve block across anesthesia groups

	Subgroups		
	**GA**	**SA**	**Total**
**Peripheral nerve block type**	***N=1352***	***N=1336***	***N=2688***
Femoral	688 (50.9)	263 (19.7)	951 (35.4)
Femoral and obturator	1 (0.1)	0 (0)	1 (0)
Femoral and saphenous	1 (0.1)	0 (0)	1 (0)
Femoral and sciatic	245 (18.1)	163 (12.2)	408 (15.2)
Obturator	1 (0.1)	0 (0)	1 (0)
Saphenous	41 (3.0)	23 (1.7)	64 (2.4)
Missing information on specific type	375 (27.7)	887 (66.4)	1262 (46.9)

Data are presented as n (%). GA=general anesthesia, SA=spinal anesthesia.
